# Association Between Dietary Advanced Glycation End Products (AGEs) and Pancreatic Fat Accumulation: Evidence From a Case–Control Study

**DOI:** 10.1002/edm2.70253

**Published:** 2026-06-03

**Authors:** Mohammad Reza Shahparvari, Zahra Yari, Maedeh Chegini, Mohammad Bahrizadeh, Amir Sadeghi, Azita Hekmatdoost

**Affiliations:** ^1^ Department of Clinical Nutrition and Dietetics, Faculty of Nutrition Sciences and Food Technology, National Nutrition and Food Technology Research Institute Shahid Beheshti University of Medical Sciences Tehran Iran; ^2^ Department of Nutrition Research, National Nutrition and Food Technology Research Institute and Faculty of Nutrition Sciences and Food Technology Shahid Beheshti University of Medical Sciences Tehran Iran; ^3^ Research Institute for Gastroenterology and Liver Diseases of Taleghani Hospital Shahid Beheshti University of Medical Sciences Tehran Iran

**Keywords:** advanced glycation end products, case–control study, diet, metabolic health, pancreatic steatosis

## Abstract

**Background:**

Pancreatic steatosis (PS) is a metabolic condition associated with obesity, insulin resistance, and fatty liver disease. Advanced Glycation End Products (AGEs), abundant in processed and high‐temperature–cooked foods, have been linked to several metabolic disorders; however, their relationship with PS has not been previously examined.

**Methods:**

In this case–control study, 278 individuals with gallstones, aged 55.7 ± 15.1 years, were classified as cases (with PS, *n* = 89) or controls (without PS, *n* = 189). PS was defined based on increased echogenicity of the pancreatic parenchyma relative to surrounding structures. Dietary intake was assessed using a food frequency questionnaire. Logistic regression models were used to estimate odds ratios (ORs) and 95% confidence intervals (CIs) for PS across quartiles of dietary AGE intake. The fully adjusted model included age, sex, total energy intake, body mass index, smoking status, and alcohol consumption.

**Results:**

Higher dietary AGE intake was significantly associated with an increased likelihood of PS. In the crude model, odds of PS did not differ significantly across quartiles of dietary AGE intake compared with the lowest quartile. After adjustment for age and sex, individuals in the highest quartile had higher odds of PS (OR = 3.56; CI: 1.1–11.6; *p* = 0.040). In the fully adjusted model, significant associations were observed for the third (OR = 2.76; CI: 1.69–11.1) and fourth (OR = 3.3; CI: 1.79–13.7) quartiles of dietary AGE intake compared with the lowest quartile. A significant positive trend in the odds of PS was observed across increasing quartiles of dietary AGE intake (p for trend = 0.024).

**Conclusion:**

Our findings suggest a potential role of dietary AGEs in pancreatic fat accumulation; however, causality cannot be inferred. Larger prospective and interventional studies are needed to confirm these associations and to determine whether reducing dietary AGE exposure can beneficially influence pancreatic fat.

## Graphical abstract

Higher dietary intake of Advanced Glycation End Products (AGEs) was associated with higher odds of pancreatic steatosis in this case–control sample of gallstone patients.

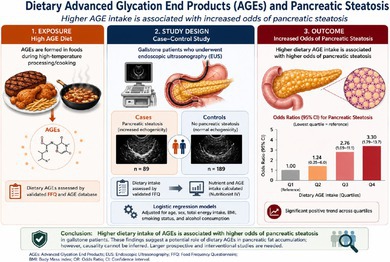

## 1 Introduction

Pancreatic steatosis refers to the abnormal accumulation of fat in the pancreas and is increasingly recognized as a prevalent condition. It shares clinical and pathological similarities with metabolic syndrome and non‐alcoholic fatty liver disease (NAFLD). Pancreatic steatosis has been linked to adverse metabolic outcomes and may be associated with an increased risk of pancreatic cancer and other pancreatic disorders [1]. Given the global burden of obesity and metabolic syndrome, pancreatic steatosis, particularly in the form of non‐alcoholic fatty pancreas disease (NAFPD), has emerged as a major concern for pancreatologists, gastroenterologists, diabetologists, and nutritionists [2, 3].

Although the exact pathogenesis of pancreatic steatosis remains unclear, several mechanisms have been proposed. In individuals with pre‐existing pancreatic disease, fat accumulation is often attributed to the replacement of acinar cells by adipose tissue. In those without underlying disease, obesity‐inducing diets and sedentary lifestyles are thought to promote ectopic fat deposition in the pancreas [1]. Other contributing factors include congenital disorders, alcohol abuse, infections, malnutrition, and metabolic syndrome. Consistently, pancreatic fat content has been positively associated with higher body mass index (BMI) and older age [2, 4]. Lifestyle and dietary patterns appear to play a pivotal role in both the development and potential improvement of this condition [2].

The modern Western diet, characterized by highly processed foods rich in fats, sugars, and salt, also contains harmful compounds known as Advanced Glycation End Products (AGEs). AGEs are formed through glycation, a process in which proteins or fats combine with sugars, either endogenously in the bloodstream or exogenously during food preparation. Cooking methods involving high temperatures, such as grilling, frying, or roasting, generate substantial amounts of AGEs, making diet the primary source of exposure [5, 6, 7, 8].

Accumulating evidence indicates that high dietary intake of AGEs and their deposition in tissues exert deleterious effects on metabolic health. AGEs are strongly linked to inflammation, oxidative stress, and insulin resistance (IR), and have been implicated in the pathogenesis of diabetes and its complications, cardiovascular diseases (CVDs), obesity, kidney disorders, and neurological conditions [9, 10, 11, 12]. Experimental and clinical data also suggest a role for dietary AGEs in NAFLD and related hepatic outcomes. Given the shared pathophysiological mechanisms between NAFLD and pancreatic steatosis, including obesity, insulin resistance, oxidative stress, and chronic low‐grade inflammation, it is plausible that dietary AGEs may also contribute to pancreatic fat accumulation [13]. Despite this biological plausibility, the direct relationship between dietary AGEs and pancreatic steatosis has not been thoroughly investigated in human populations. This gap in knowledge underscores the need for studies examining whether higher dietary AGE intake is associated with pancreatic fat accumulation. Therefore, the present study aimed to examine the association between dietary intake of AGEs and the odds of pancreatic steatosis in a case–control sample of adult patients undergoing endoscopic ultrasonography.

## 2 Methods and Materials

### 2.1 Study Design and Participants

This case–control study was conducted among adult patients with gallstone disease who were referred to the gastroenterology clinic of Taleghani Hospital, Shahid Beheshti University of Medical Sciences, Tehran, Iran. All participants underwent endoscopic ultrasonography as part of their routine diagnostic evaluation. The overall study protocol has been described previously [14]; however, the essential methodological details relevant to the present analysis are summarized here.

### 2.2 Data Collection and Measurements

In total, 278 participants were included in the analysis, comprising 89 cases with pancreatic steatosis and 189 controls without pancreatic steatosis. Demographic and lifestyle information (age, sex, education, marital status, smoking, alcohol use, supplement/medication use, and medical history) was obtained using a structured questionnaire. Anthropometric indices, including weight, height, BMI, and waist circumference, were measured following standard protocols.

### 2.3 Assessment of Pancreatic Steatosis

Pancreatic steatosis was assessed by endoscopic ultrasonography performed by experienced gastroenterologists who were blinded to the dietary data. The pancreas was systematically examined in standard views. Pancreatic steatosis was defined as increased echogenicity of the pancreatic parenchyma compared with the surrounding soft tissue and adjacent structures (e.g., spleen or kidney), with preserved glandular architecture. When applicable, steatosis was graded qualitatively (absent, mild, moderate, or severe) based on the degree of increased echogenicity and attenuation. For the purposes of this analysis, any degree of pancreatic steatosis (mild, moderate, or severe) was considered as presence of steatosis, and these individuals were classified as cases. Participants with no visible evidence of increased echogenicity or other sonographic features suggestive of steatosis were classified as controls.

### 2.4 Dietary Assessment and AGE Calculation

Dietary intake was assessed using a validated semi‐quantitative Food Frequency Questionnaire (FFQ) specifically adapted for the Iranian population. The FFQ was administered in face‐to‐face interviews by trained dietitians. Standard portion sizes were explained to participants, and they reported the frequency of consumption of each food item over the past year. Nutrient intake, including total energy, macronutrients, and food groups, was calculated using Nutritionist IV software.

Because the Iranian Food Composition Table does not provide AGE values, dietary AGE intake was estimated using the U.S. food AGE database [15, 16], which reports AGE content for 549 commonly consumed food items measured by a validated immunoassay (ELISA‐based method targeting Nε‐carboxymethyl‐lysine, a major AGE marker). AGE values were expressed in kilo‐units (kU) per 100 g of solid food or 100 mL of liquid food. Reported household measures were converted into grams, and the daily AGE intake for each participant was calculated by multiplying the AGE content of each food item by the reported frequency and portion size, then summing across all foods.

When an exact match for a given food item was not available in the AGE database, we used AGE values from comparable foods with similar composition and cooking methods. Although this approach improves coverage of the database, it may not fully capture differences in local recipes and preparation techniques. The FFQ did not collect detailed information on cooking methods (e.g., grilling, frying, baking, boiling), cooking temperature, or duration for each food item, which we acknowledge as an important limitation discussed below.

### 2.5 Ethics

All participants provided written informed consent. The Ethics Committee of National Nutrition and Food Technology Research Institute, Shahid Beheshti University of Medical Sciences (IR.SBMU.NNFTRI.REC.1403.074) approved the study protocol. All procedures were conducted in accordance with the Declaration of Helsinki.

### 2.6 Statistical Analysis

Baseline characteristics were summarized as mean ± SD for continuous variables and percentages for categorical variables. Differences between groups were assessed using ANOVA and chi‐square tests. Participants were categorized into quartiles of total dietary AGE intake, with the lowest quartile serving as the reference group. Logistic regression models were used to estimate odds ratios (ORs) and 95% confidence intervals (CIs) for the association between dietary AGE quartiles and the presence of pancreatic steatosis. In Model 1 (crude), no covariates were included. In Model 2, we adjusted for age and sex. In Model 3, we additionally adjusted for total energy intake, BMI, smoking status, and alcohol consumption, as major potential confounders identified a priori. To assess linear trends across quartiles of dietary AGE intake, we assigned the median AGE value of each quartile to participants in that category and included this variable as a continuous term in the logistic regression models; the corresponding *p*‐value was reported as p for trend. All statistical analyses were performed using SPSS software, version 22 (SPSS Inc., Chicago, IL, USA). A two‐sided *p*‐value < 0.05 was considered statistically significant.

## 3 Results

Table 1 summarizes the baseline characteristics of participants across quartiles of dietary AGE intake. A higher intake of AGEs was significantly associated with male sex (*p* = 0.006), alcohol consumption (*p* = 0.032), and smoking (*p* = 0.017). No significant differences were observed in age, weight, height, BMI, or other anthropometric indices (*p* > 0.05).

**TABLE 1:** Characteristics of study participants by quartiles of advanced glycation end products (AGEs).

	Quartile of total AGEs				
	Q1 < 7906	Q2 7905–11,039	Q3 11,039–15,471	Q4 > 15,471	*p*
Men, %	55.6	51.7	73.3	88.2	0.006
Age (y)	54.6 ± 10.1	53.2 ± 10.5	55.6 ± 12.7	55.4 ± 13.9	0.871
Alcohol drinker	26.9	28.6	3.6	34.4	0.032
Smoker, %	30.8	31	32.1	63.6	0.017
Weight, kg	75.6 ± 19.2	76.8 ± 17.8	71.2 ± 12.2	72.9 ± 16	0.556
Height, cm	163.7 ± 8.5	163.7 ± 8.9	167.3 ± 8.2	167 ± 6.7	0.141
Body mass index, kg/m^2^	28.2 ± 6.1	28.8 ± 5.6	25.8 ± 4.4	26.2 ± 4.8	0.076
Waist circumference (cm)					

*Note:* Values are means ± SDs for continuous variables and percentages for categorical variables. ANOVA for quantitative variables and *χ* ^2^ test for qualitative variables.

Table 2 presents dietary intake across AGE quartiles. Individuals in higher AGE quartiles consumed significantly more total energy (*p* = 0.016) and meat products, which are major dietary sources of AGEs (*p* = 0.001). A decreasing trend in carbohydrate intake was observed with increasing AGE intake, approaching statistical significance (*p* = 0.052). No significant associations were found between AGE intake and other food groups, including vegetables, fruits, cereals, dairy, or fiber (*p* > 0.05).

**TABLE 2:** Dietary intakes of study participants by quartiles of advanced glycation end products (AGEs).

	Quartile of total AGEs				
	Q1	Q2	Q3	Q4	*p*
Energy (kcal/d)	1851 ± 653	2102 ± 628	2165 ± 445	2427 ± 736	0.016
Protein (% TEI)	13.9 ± 2.4	14.3 ± 2.5	15.2 ± 2.9	15 ± 3.4	0.336
Carbohydrate (% TEI)	64.8 ± 9.7	61.2 ± 6.4	60 ± 6.2	59.5 ± 5.4	0.052
Fat (% TEI)	27.7 ± 6.6	29.2 ± 9.9	27.8 ± 5.8	28.4 ± 8.7	0.577922
Fibre (1000 g/kcal)	13.7 ± 3.7	15.4 ± 3.5	14.8 ± 43.8	14.5 ± 4.1	0.510
Vegetables	209.2 ± 144.3	202.5 ± 133.7	235.1 ± 165.3	257.9 ± 167.7	0.509
Fruits	351.6 ± 228.7	406.9 ± 195.8	356.8 ± 216.4	410.1 ± 173.8	0.620
Meats	35.4 ± 15.4	63.1 ± 26.3	93 ± 43.3	96.5 ± 49.5	0.001
Cereals	134.2 ± 88.4	157.6 ± 118.4	182.4 ± 151.7	185.7 ± 130.1	0.381
Dairy	232.2 ± 159.3	241.3 ± 143.6	250.5 ± 179.6	271.3 ± 149.9	0.841

*Note:* Values are means ± SDs for continuous variables and percentages for categorical variables. ANOVA for quantitative variables and *χ* ^2^ test for qualitative variables.

**FIGURE 1:**
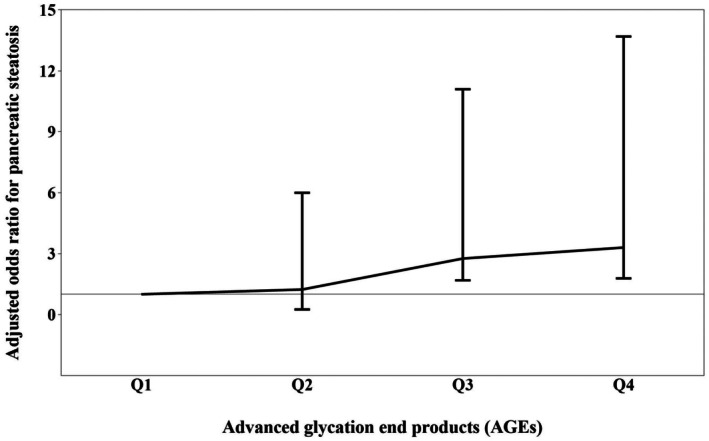
The trend of the risk of pancreatic steatosis with increasing the intake of advanced glycation end products.

Table 3 shows the odds ratios (ORs) and 95% confidence intervals (CIs) for pancreatic steatosis across AGE quartiles. The first quartile served as the reference group. In the crude model, no significant association was observed (*p* > 0.05). In Model 2, adjusted for age and sex, a significant association was found between the fourth quartile of AGE intake and pancreatic steatosis (OR = 3.56, 95% CI: 1.1–11.6; *p* = 0.040). In Model 3, further adjusted for energy intake, BMI, smoking, and alcohol consumption, significant associations were observed for both the third and fourth quartiles compared to the reference group (OR = 2.76, 95% CI: 1.69–11.1; OR = 3.3, 95% CI: 1.79–13.7; *p* = 0.048). Overall, a significant positive trend was observed between increasing dietary AGE intake and the likelihood of pancreatic steatosis (p‐trend = 0.024). Figure 1 illustrates the increasing trend in pancreatic steatosis prevalence across AGE intake quartiles, highlighting a significant rise in risk with higher dietary AGE exposure.

**TABLE 3:** Odds ratios (OR) and 95% confidence intervals (CI) for occurrence of the PS in each quartiles of the advanced glycation end products (AGEs).

AGEs	Q1	Q2	Q3	Q4	*p* trend
No. of deaths	5	9	12	17	0.024
Model 1	ref	2.1 (0.69–6.2)	2.47 (0.85–7.1)	2.64 (0.9–7.5)	0.075
Model 2	ref	2.54 (0.81–8)	2.75 (0.79–9.5)	3.56 (1.1–11.6)	0.040
Model 3	ref	1.24 (0.25–6)	2.76 (1.69–11.1)	3.3 (1.79–13.7)	0.048

*Note:* Based on multiple logistic regression model. Model 1: crude. Model 2: adjusted for age and sex. Model 3: additionally adjusted for energy intake, BMI, smoking, alcohol.

## 4 Discussion

The present study is the first to investigate the association between dietary intake of AGEs and the risk of pancreatic steatosis. Our findings demonstrate that higher dietary AGE intake is significantly associated with an increased likelihood of developing pancreatic steatosis. Participants in the higher AGE quartiles were more likely to be men, smokers, and alcohol consumers, and they also exhibited higher total energy intake and greater consumption of meat products, one of the richest dietary sources of AGEs. Conversely, carbohydrate intake showed a decreasing trend with increasing AGE intake.

Although no previous study has directly examined the relationship between AGEs and pancreatic steatosis, our findings align with a growing body of evidence linking AGEs to metabolic and hepatic disorders. LiJiao et al. reported that higher intake of CML‐AGEs, particularly from red meat, increased the risk of pancreatic cancer in men [17]. Fernando et al. identified AGEs as a key driver in the progression of hepatic steatosis to non‐alcoholic steatohepatitis (NASH) [18]. Similarly, elevated circulating or tissue levels of AGEs have been reported in both NAFLD and alcoholic liver disease [19]. In addition, higher dietary AGE intake has been associated with NAFLD occurrence [13] and with increased mortality risk in patients with cirrhosis [20]. Additional evidence from Mendoza‐Herrera et al. indicated that higher AGE intake was associated with elevated fasting glucose and metabolic syndrome in young adults [21]. Furthermore, a low‐AGE diet has been shown to improve insulin resistance in overweight women [22]. However, Angoorani et al. reported that although high AGE intake was associated with abdominal obesity and elevated triglycerides, it was not significantly associated with metabolic syndrome [23].

Collectively, these findings support the hypothesis that AGEs contribute to metabolic dysfunction and ectopic fat accumulation. The biological plausibility of this association is well established. AGEs exert their pathogenic effects primarily through binding to the Receptor for Advanced Glycation End Products (RAGE). Activation of RAGE triggers downstream signalling pathways such as NF‐κB and MAPK, leading to increased production of pro‐inflammatory cytokines including TNF‐α and IL‐6, which promote chronic low‐grade inflammation [18]. AGE–RAGE interaction also enhances the generation of reactive oxygen species (ROS), contributing to oxidative stress and cellular injury [24]. These changes promote chronic low‐grade inflammation and oxidative stress, which may exacerbate insulin resistance and favor ectopic lipid deposition in organs such as the liver and pancreas [25, 26]. AGE accumulation in pancreatic islets has also been shown to induce oxidative stress and β‐cell injury, potentially impairing insulin secretion and further aggravating metabolic disturbances [27]. While these mechanisms provide biological plausibility for a link between dietary AGEs and pancreatic fat, our observational data cannot establish causality.

In interpreting our findings, it is important to consider potential confounding. Participants with higher dietary AGE intake also tended to have higher total energy intake, greater consumption of meat products, and a higher prevalence of smoking and alcohol use. We adjusted for age, sex, total energy intake, BMI, smoking status, and alcohol consumption in multivariable models, which strengthened the associations between higher AGE quartiles and pancreatic steatosis compared with the crude model. Nonetheless, residual confounding by unmeasured or imperfectly measured lifestyle factors cannot be excluded. We did not collect comprehensive data on physical activity, socioeconomic status, or detailed medication use, and information on some comorbidities may have been incomplete. These factors may be associated with both dietary AGE intake and pancreatic fat accumulation and could influence the observed associations.

This study has several strengths. First, it addresses a novel research question by examining dietary AGEs in relation to pancreatic steatosis, thereby filling an important gap in the literature. Second, pancreatic steatosis was assessed using endoscopic ultrasonography, a sensitive clinical method performed by experienced gastroenterologists, which enhances the validity of outcome classification. Third, data on diet and covariates were collected using standardized instruments and by trained interviewers, helping to minimize interviewer bias and improve internal consistency. Fourth, we used a validated food frequency questionnaire combined with a comprehensive AGE database to estimate dietary AGE intake, enabling us to examine dose–response patterns across quartiles of exposure.

At the same time, several limitations should be acknowledged. The case–control design precludes causal inference and does not allow for the assessment of temporal relationships between dietary AGE intake and the development of pancreatic steatosis. The relatively wide confidence intervals around some of the odds ratio estimates indicate limited precision, suggesting that our results should be interpreted with caution. All participants were recruited among patients with gallstone disease undergoing endoscopic ultrasonography at a single tertiary care center, which may limit the generalizability of the findings to other clinical populations or to the general population. The relationship between dietary AGEs and pancreatic steatosis may differ in individuals without gallstones or in other settings.

In addition, dietary intake was assessed using a semi‐quantitative food frequency questionnaire, which is inherently prone to recall error and misreporting. The AGE content of foods was derived from a U.S.‐based database, which may not perfectly reflect Iranian food items, recipes, or cooking practices. Furthermore, the questionnaire did not capture detailed information on cooking methods, temperature, or duration for each food item, even though these factors are major determinants of AGE formation. These issues likely resulted in misclassification of individual AGE intakes. Such misclassification is expected to be largely non‐differential with respect to case–control status, and would therefore tend to bias associations toward the null rather than create spurious positive associations. We also lacked detailed data on physical activity, socioeconomic status, and comprehensive medication use, and serum or tissue AGE levels were not measured, limiting our ability to explore mechanistic pathways more directly.

Despite these limitations, the present study provides preliminary evidence that higher dietary AGE intake is associated with higher odds of pancreatic steatosis in adult gallstone patients. These findings, together with prior work on AGEs and other metabolic outcomes, support the need for further research on the potential role of dietary AGEs in pancreatic health. Prospective cohort studies and randomized controlled trials incorporating detailed assessment of cooking methods, objective measures of AGE exposure, and more diverse populations will be essential to confirm our observations and to determine whether reducing dietary AGE intake can beneficially affect pancreatic fat content and related metabolic outcomes.

## 5 Conclusion

In this case–control study of adult gallstone patients undergoing endoscopic ultrasonography, higher dietary intake of advanced glycation end products was associated with higher odds of pancreatic steatosis. While these findings suggest a potential role for dietary AGEs in pancreatic fat accumulation, the observational design, limited precision of the estimates, and potential residual confounding preclude causal inferences. Larger prospective and interventional studies in more diverse populations are needed to confirm these associations and to clarify whether reducing dietary AGE exposure can beneficially influence pancreatic fat and related metabolic outcomes.

## Author Contributions

**Zahra Yari:** conceptualization, formal analysis, writing – original draft, writing – review and editing. **Mohammad Bahrizadeh:** methodology. **Maedeh Chegini:** methodology. **Mohammad Reza Shahparvari:** project administration, writing – original draft. **Azita Hekmatdoost:** conceptualization, writing – review and editing. **Amir Sadeghi:** methodology.

## Funding

This work was supported by Shahid Beheshti University of Medical Sciences (143132).

## Ethics Statement

The Research Ethics Committees of Ethics Committee of National Nutrition and Food Technology Research Institute, Shahid Beheshti University of Medical Sciences reviewed and approved the study methods and design (IR.SBMU.NNFTRI.REC.1403.074).

## Conflicts of Interest

The authors declare no conflicts of interest.

## Data Availability

The data that support the findings of this study are available from the corresponding author upon reasonable request.
